# Retrospectively self-reported age of childhood abuse onset in a United States nationally representative sample

**DOI:** 10.1186/s40621-017-0103-1

**Published:** 2017-03-13

**Authors:** Alison L. Cammack, Carol J. Hogue

**Affiliations:** Department of Epidemiology, Emory University, Rollins School of Public Health, 1518 Clifton Rd NE, Atlanta, GA 30322 USA

**Keywords:** Child abuse, Sexual abuse, Sexual assault, Physical abuse, Child maltreatment, Trauma

## Abstract

**Background:**

Child abuse is common and several studies have linked it to health outcomes throughout the lifecourse. Recent information about timing of abuse reported retrospectively is underrepresented in the literature, despite its importance to informing target populations for primary prevention of child abuse and studying effects of child abuse. ﻿This study uses data from Wave IV (2008–2009) of The National Longitudinal Study of Adolescent Health to Adult Health (N = 14,776) to describe distributions of retrospectively self-reported age of onset of childhood emotional, physical, and sexual abuse perpetrated by parents/adult caregivers and sexual abuse perpetrated by other individuals. Information on childhood abuse history was collected when participants were between 24 and 32 years old.

**Findings:**

Parental/adult caregiver perpetrated abuse frequently started in early childhood, particularly sexual abuse. Non-parental/adult caregiver sexual abuse motivated by physical force also started early in boys (median age = 7.21 years (95% CI: 5.92, 9.05)). Earlier onset of some types of abuse was associated with male sex, not being raised by both biological parents, and low childhood household income.

**Conclusions:**

Future studies should further examine timing of childhood abuse onset and include diverse measures of abuse, including those derived from longitudinal studies and validated reports. If these results are replicated, they suggest that abuse, particularly sexual abuse perpetrated by parents/adult caregivers, often starts in early childhood, and preventive interventions should be designed to protect younger children.

## Background

Adults commonly report having been abused as children, and several studies have linked childhood abuse to various mental and physical outcomes from childhood through late life (Shonkoff and Garner 2012). In samples with good generalizability (e.g., those that used population-based sampling), the prevalence of adult self-reported childhood abuse history ranges from 11–37% for emotional abuse, 11–15% for physical abuse, and 6–54% for sexual abuse, with more women than men reporting history of sexual abuse (Finkelhor 1994; Gorey and Leslie 1997; Felitti et al. 1998; Centers for Disease Control and Prevention (CDC) 2010). For some outcomes, the etiologic fraction that is attributable to adverse childhood experiences, including abuse, is particularly high. Afifi (Afifi et al. 2008) reported that up to half of suicide attempts are attributable to childhood adversity.

While many instruments assess retrospective self-reported childhood abuse history (Felitti et al. 1998; Bernstein et al. 2003), except for a recently developed measure (Teicher and Parigger 2015), these measures seldom collect detailed information about timing of abuse onset. Further, despite many epidemiologic studies examining the frequency of abuse via retrospective self-report (Hovdestad et al. 2015), usually only limited data about age of abuse onset are captured through this method. For example, age of first rape is described in the National Violence Against Women Survey (NVAWS), a nationally representative survey of women and men (Tjaden and Thoennes 2000), and information about timing of sexual abuse is available from a nationally representative population-based study conducted in 1985 (Finkelhor et al. 1990), but timing of other forms of childhood abuse, such as physical or emotional abuse, are not included in these assessments. More recent data of timing of abuse onset can be obtained through other methods, including reported abuse cases (e.g., Child Protective Service records) (Administration on Children, Youth and Families, Children’s Bureau 2012) and studies that incorporate longitudinal study designs (e.g., Longitudinal Studies on Child Abuse and Neglect) (Runyan et al. 1998).

Although retrospective self-report is a method that is convenient to use in large population-based samples, allowing for assessments that yield good generalizability, it is subject to recall error which compromises internal validity. Some studies have found that a substantial percentage of individuals with documented abuse may not recall abuse as adults (Widom and Shepard 1996; Widom and Morris 1997). However, this source of bias must be balanced against the fact that retrospective studies capture individuals who may not be identified in longitudinal studies, which frequently utilize at-risk populations, and/or those not identified as victims in the criminal justice system. Substantiated abuse only captures a small proportion of truly exposed individuals, and identified children may systematically differ from the overall population of individuals exposed to maltreatment (Gilbert et al. 2009). Some studies have also noted racial biases in substantiated abuse (Dettlaff et al. 2011). In some studies that utilize both longitudinal and retrospective measures of abuse, more individuals report abuse retrospectively (Kendall-Tackett and Becker-Blease 2004). Finally, unlike retrospective studies of adults, longitudinal studies may have compromised internal validity because required reporting may affect willingness to disclose abuse (Amaya-Jackson et al. 2000). Thus, in spite of limitations to retrospective self-report, others argue that measurement of serious abuse using this method has a worthwhile and important place in the literature, and it may yield information about abuse from individuals that are not captured through other methods (Kendall-Tackett and Becker-Blease 2004; Hardt and Rutter 2004).

Information about age of abuse onset is important for determining target populations for prevention and identification-based interventions. Age of abuse onset may also be an important factor in determining effects of maltreatment. Evidence from the developmental literature stresses the presence of exposure windows of increased susceptibility to the effects of harmful environments (Wachs et al. 2014), and timing of abuse has been specifically linked to severity of subsequent psychopathology in adulthood (Kaplow and Widom 2007). Thus, to examine more recent retrospective self-reports and to expand the number of abuse types examined via this method, the goal of this paper was to describe the age of onset of multiple types of retrospectively self-reported abuse in a nationally representative sample.

## Methods

The National Longitudinal Study of Adolescent to Adult Health (“Add Health”) (Harris et al. 2009) interviewed adolescents in grades 7 through 12 during 1994–95 in schools located in 80 communities throughout the United States. Sampling methods and stratification ensured that selected schools were representative of US schools with respect to region of country, urbanicity, school size and type, and race/ethnicity. A subset of participants were followed through ages 24–32 in 2008–2009 (*n* = 15,701; response rate = 80.3%), when they were queried about history of childhood abuse. Inclusion criteria for the present study (*N* = 14,776) were 1) presence of a Wave IV sampling weight; 2) data for any of the childhood abuse questions in Wave IV. Permission to conduct secondary analyses was approved by the Emory University Institutional Review Board.

### Measures

Abuse history was assessed via five questions through a computer-assisted self interview. Three questions assessed parental/adult caregiver sexual, physical, and emotional abuse, and two questions assessed non-parental/adult caregiver sexual abuse motivated by physical force and non-physical threats (Harris et al. 2009; Briere and Elliott 2003; Crowley et al. 2003; Straus et al. 1998). Parental/adult caregiver abuse was asked by the questions, “Before your 18th birthday, how often did a parent or other adult caregiver say things that really hurt your feelings or made you feel like you were not wanted or loved?”, “Before your 18^th^ birthday, how often did a parent or adult caregiver hit you with a fist, kick you or throw you down on the floor, into a wall, or down stairs?”, and “Before your 18^th^ birthday, how often did a parent or other adult caregiver touch you in a sexual way, force you to touch him or her in a sexual way, or force you to have sexual relations?” Questions about non-parental/adult caregiver sexual abuse were phrased as, “Have you ever been forced, in a non-physical way, to have any type of sexual activity against your will? For example, through verbal pressure, threats of harm, or by being given alcohol or drugs? Do not include any experiences with a parent or adult caregiver”, and “Have you ever been physically forced to have any type of sexual activity against your will? Do not include any experiences with a parent or adult caregiver”. For non-parental/caregiver abuse, we defined abuse as occurring before age 18. Participants were asked how old they were in continuous years when each type of abuse started (“How old were you the first or only time this happened?").

Participants were asked to categorize frequency of parental/caregiver abuse (0, 1, 2, 3-5, 6-10, or more than 10 times), and we dichotomized abuse into present or absent by selecting cut points approximating prevalences in the Adverse Childhood Experiences questionnaire in a multi-state, population based sample (Centers for Disease Control and Prevention (CDC) 2010). For these data, the cutpoints were 1 or more total times for sexual abuse (both parental/adult caregiver and non-parental/adult caregiver subtypes), 2 or more times for physical abuse, and 3 or more times for emotional abuse.

### Statistical analysis

The distribution of age at abuse onset was examined as a continuous variable, with means and percentiles for individual abuse subtypes, including 95% confidence intervals (CIs). For the subset of individuals who experienced abuse and who had information available for all potential determinants of abuse timing (*N* = 2,694, 1,321, and 528 for parent/caregiver emotional, physical, and sexual abuse, respectively, and 770 and 565 for non-parent/caregiver sexual abuse motivated by non-physical threats and physical force, respectively), we used multivariate linear regression to model age of onset of each individual abuse type, examining the independent effects of sex, race, childhood household income in 1994 as measured by parent interview (overall response rate for this question = 74% of participants with baseline Wave I data), and whether one was raised by both biological parents. All analyses incorporated complex weighting to account for the survey design using SAS callable SUDAAN (SUDAAN 11.0.1. Research Triangle Institute, Raleigh, North Carolina; SAS 9.4, SAS Institute, Cary, North Carolina) following guidelines by Chen and Chantala (Chen and Chantala 2006).

## Findings

Six thousand nine hundred nine ten males and 7857 females (unweighted numbers) participated in the survey. Table 1 shows the descriptive characteristics of the study population. The weighted sample reflected the racial/ethnic distribution of the United States. In the study population, abuse was relatively common, and sexual abuse (all types) was more common in girls than boys.

**Table 1:** Study population characteristics

Characteristic	N^a^	Total population weighted percent (95% CI)	Male population weighted percent (95% CI)	Female population weighted percent (95% CI)
Age at Wave 4 Interview	14776	Mean = 28.83(28.60,29.06)	Mean = 28.93(28.69,29.17)	Mean = 28.73(28.50,28.96)
Male	6919	50.72 (49.50,51.95)		
Female	7857	49.28 (48.05,50.50)		
Parental/Adult Caregiver Abuse				
Emotional Abuse^b^	3575	25.61(24.42,26.85)	20.48 (19.03,22.01)	30.91 (29.22,32.65)
Physical Abuse^c^	1771	11.96 (11.06,12.92)	11.85 (10.67,13.14)	12.07 (10.95,13.29)
Sexual Abuse^d^	724	4.88 (4.37,5.45)	2.49 (1.97,3.14)	7.34 (6.52,8.26)
Non-Parental/Adult Caregiver Sexual Abuse				
Motivated by Non-Physical Threat^e^	998	7.25 (6.63,7.94)	2.27 (1.81,2.84)	12.40 (11.26,13.64)
Motivated by Physical Force^f^	731	5.13 (4.65,5.65)	1.45 (1.11,1.90)	8.92 (8.01,9.93)
Race/Ethnicity^g^				
Hispanic	2352	11.93 (8.97,15.71)	11.99 (8.93,15.90)	11.88 (8.87,15.73)
Black	3213	16.02 (12.31,20.57)	15.66 (11.88,20.36)	16.38 (12.61,21.00)
White	7871	65.77 (59.86,71.23)	65.78 (59.70,71.38)	65.76 (59.77,71.29)
Asian	937	3.37 (2.15,5.24)	3.42 (2.16,5.38)	3.31 (2.07,5.26)
Native American	262	1.94 (1.49,2.53)	2.12 (1.58,2.85)	1.76 (1.27,2.43)
Other	129	0.97 (0.72,1.30)	1.03 (0.67,1.57)	0.90 (0.63,1.29)
Childhood Household Income (1994)^g^				
< 35000 per year	4827	43.50 (39.47,47.61)	43.00 (38.64,47.48)	44.02 (39.97,48.14)
= > 35000 per year	6373	56.50 (52.39,60.53)	57.00 (52.52,61.36)	55.98 (51.86,60.03)
Childhood Household Composition^g^				
Not Raised by Both Biological Parents	4493	28.74 (26.53,31.07)	27.50 (25.06,30.09)	30.02 (27.44,32.73)
Raised by Both Biological Parents	10272	71.26 (68.93,73.47)	72.50 (69.91,74.94)	69.98 (67.27,72.56)
Marital Status at Wave 4 Interview^g^				
Never Married	7375	50.20 (47.46,52.93)	55.01 (52.08,57.90)	45.24 (42.21,48.31)
Ever Married	7390	49.80 (47.07,52.54)	44.99 (42.10,47.92)	54.76 (51.69,57.79)

^a^Unweighted Sample Size

^b^594 subjects with missing data

^c^312 subjects with missing data

^d^152 subjects with missing data

^e^65 subjects with missing data

^f^44 subjects with missing data

^g^Subheadings for variables (other than abuse) may not total to 14,776 due to missing data

Table 2 describes the population estimated distribution of age of onset of abuse by sex. The distribution of parental/adult caregiver sexual abuse was shifted towards particularly early ages; medians indicated this type of abuse occurred for most individuals before age 8, and for more than a quarter of those affected, abuse started before the typical age of entry into elementary school. By contrast, the median age of onset for non-parental/adult caregiver sexual abuse experienced by girls indicated that it occurred for most at a later age than parental/caregiver abuse, most commonly during adolescence. In boys, however, the difference between non-parental/adult caregiver versus parent/adult caregiver perpetrated sexual abuse was less defined, particularly for non-parental abuse motivated by physical force. Median age of onset indicated that physical and emotional abuse typically started before adolescence for both sexes.

**Table 2:** Population Weighted Age Distribution of Childhood Abuse Onset by Sex

	N^a^	Mean (95% CI)	Median (95% CI)	10th Percentile (95% CI)	25th Percentile (95% CI)	75th Percentile (95% CI)	90th Percentile (95% CI)
Boys							
Parent/Adult Caregiver Abuse							
Emotional Abuse	1337	9.57 (9.30,9.85)	9.25 (8.92,9.53)	4.18 (3.89,4.45)	5.88 (5.49,6.36)	12.04 (11.74,12.45)	14.36 (13.93,14.69)
Physical Abuse	856	9.17 (8.76,9.59)	8.37 (7.62,9.30)	3.77 (3.14,4.20)	5.21 (4.81,5.74)	11.94 (11.54,12.55)	14.32 (13.79,14.78)
Sexual Abuse	168	7.78 (6.93,8.63)	6.36 (5.50,7.04)	1.48 (0.66,3.37)	4.28 (3.51,4.93)	10.37 (8.16,12.20)	14.57 (12.13,-^b^)
Non- Parental/Adult Caregiver Sexual Abuse							
Motivated by Non-Physical Threat	148	10.66 (9.66,11.66)	10.43 (7.91,12.41)	4.46 (3.95,5.23)	5.81 (5.15,7.20)	14.51 (13.05,15.35)	15.91 (14.96,-^b^)
Motivated by Physical Force	102	8.48 (7.60,9.37)	7.21 (5.92,9.05)	2.83 (1.46,4.52)	4.97 (4.32,5.60)	11.72 (9.25,13.30)	14.12 (12.38,15.41)
Girls							
Parent/Adult Caregiver Abuse							
Emotional Abuse	2238	10.07 (9.81,10.34)	9.92 (9.54,10.75)	4.11 (3.85,4.29)	5.96 (5.61,6.42)	12.96 (12.67,13.29)	14.76 (14.57,14.93)
Physical Abuse	915	9.89 (9.55,10.23)	9.46 (8.86,9.99)	4.15 (3.83,4.38)	5.74 (5.22,6.27)	12.99 (12.53,13.48)	14.81 (14.50,15.08)
Sexual Abuse	556	8.18 (7.80,8.56)	7.51 (6.87,8.10)	2.69 (2.35,3.16)	4.45 (4.12,4.82)	10.59 (9.79,11.20)	13.33 (12.52,13.95)
Non- Parental/Adult Caregiver Sexual Abuse							
Motivated by Non-Physical Threat	850	12.68 (12.31,13.06)	13.76 (13.32,14.12)	4.89 (4.42,5.85)	10.01 (8.55,11.15)	15.46 (15.22,15.68)	16.40 (-^b^, -^b^)
Motivated by Physical Force	629	12.52 (12.12,12.93)	13.47 (12.82,14.08)	5.27 (4.62,6.31)	8.85 (7.95,10.28)	15.37 (15.08,15.62)	16.32 (-^b^, -^b^)

^a^Unweighted Sample Size

^b^Value not computed (Denominator of the interpolation is zero)

In regression models (Table 3), except for parental/caregiver sexual abuse, abuse occurred significantly and in some cases substantially earlier in boys than girls. Most other statistically significant effect sizes translated to approximately a one year age difference, compared to unexposed individuals. Childhood household income below $35,000 per year was associated with significantly earlier onset of non-parental/caregiver sexual abuse motivated by non-physical threats and physical force. Not being raised by both biological parents was associated with significantly earlier emotional and physical abuse. Compared to non-Hispanic whites, Black race was broadly associated with later onset of abuse. A sensitivity analysis utilizing log transformed age of abuse onset yielded similar associations.

**Table 3:** Linear Regression for Age of Childhood Abuse Onset: Beta Coefficients for Independent Predictors

	Hispanic versus White (referent) Beta (95% CI)	Black versus White (referent) Beta (95% CI)	Other Race^a^versus White (referent) Beta (95% CI)	Childhood Household Income: >$35,000 Per Year versus < = $35,000 Per Year (referent) Beta (95% CI)	Not Raised by Both Biological Parents versus Raised by Both Biological Parents (referent) Beta (95% CI)	Male versus Female (referent) Beta (95% CI)
Parental/Adult Caregiver Abuse						
Emotional Abuse	1.00 (0.36, 1.63)^b^	1.25 (0.60, 1.90)^b^	-0.74 (-1.71, 0.24)	0.20 (-0.19, 0.60)	-0.94 (-1.33, -0.54)^b^	-0.67 (-1.07, -0.27)^b^
Physical Abuse	-0.45 (-1.29, 0.39)	0.85 (0.08, 1.61)^b^	-0.94 (-2.07, 0.19)	0.06 (-0.54, 0.65)	-0.80 (-1.41, -0.19)^b^	-0.69 (-1.33, -0.06)^b^
Sexual Abuse	-0.62 (-1.82, 0.59)	0.95 (-0.38, 2.28)	0.60 (-0.83, 2.03)	0.52 (-0.35, 1.39)	0.22 (-0.64, 1.09)	-0.67 (-1.79, 0.44)
Non- Parental/Adult Caregiver Sexual Abuse						
Motivated by Non-Physical Threat	-0.87 (-2.10, 0.36)	0.30 (-1.03, 1.62)	-0.64 (-2.64, 1.37)	-0.90 (-1.65, -0.14)^b^	0.13 (-0.62, 0.88)	-2.37 (-3.48, -1.26)^b^
Motivated by Physical Force	0.62 (-0.60, 1.85)	0.18 (-0.88, 1.25)	-1.77 (-3.74, 0.19)	-1.12 (-1.95, -0.29)^b^	-0.15 (-1.13, 0.82)	-4.00 (-5.06, -2.95)^b^

^a^Due to small sample size, this variable combined Asian, Native American, and Other races

^b^(*p* < 0.05)

## Discussion

This study adds to the literature describing age of various types of childhood abuse onset via retrospective-self report, a measure that has been underutilized in recent studies, in a nationally representative sample. In this sample, sexual abuse perpetrated by parents/adult caregivers began particularly early. The median onset of sexual abuse perpetrated by parents/adult caregivers is notably younger than that found in older surveys from the 1980s (median ages between 10 and 11 years) (Finkelhor 1986), underscoring the vulnerability of children experiencing this form of victimization and the importance of considering the role of the perpetrator. Our finding of boys reporting some types of sexual abuse on average at an earlier age than girls is consistent with data from the NVAWS, which reported that among boys experiencing rape, 48% experienced it before age 12, versus 24% in girls (Tjaden and Thoennes 2000). Also, parent/caregiver sexual abuse occurring earlier than physical abuse is consistent with previous, limited analyses of these data (Dunn et al. 2013). However, trends for sexual abuse occurring particularly early differ from data relying on reported abuse, where most physical and emotional abuse occurred before age 9, but most sexual abuse occurred after age 9 (Children and Youth and Families Children’s Bureau. Childhood Maltreatment 2012). Some have reported that longitudinal child or parent reports of all types of abuse tend to increase with age (Gilbert et al. 2009), which differs from some, but not all of our findings.

The effect of interventions for the prevention of child abuse remains unclear, so there is a need for accurate information on characteristics of abuse, such as age of onset, to guide the development of impactful interventions. For example, several interventions shown to increase children’s knowledge of abuse and protective behaviors are based in the school setting (Macmillan et al. 2009; Walsh et al. 2015), but these findings suggest that abuse, particularly sexual abuse, may begin before elementary school. This suggests that preventive interventions for early childcare providers and parents may be important to reducing sexual abuse. Also, fewer interventions exist for physical and emotional abuse in school settings (Macmillan et al. 2009); these data suggest that school based interventions for prevention of these exposures may reach many children.

An important limitation of our study, as previously mentioned, is that retrospective self-report may be prone to recall error. Research indicates that adults have the ability to recall events from much of their childhood (Tustin and Hayne 2010), but the extent to which details like timing can be accurately recalled is unclear. Very early abuse may be underreported due to limitations in memory, which implies that the true distribution of abuse onset is shifted to earlier ages. However, we note that these data were collected in early adulthood, which may have reduced misreporting related to length of time since abuse occurred. Another limitation is that the Add Health study population is representative of students enrolled in grades 7–12 and is not generalizable to children who have dropped out of school, although Add Health did sample continuation schools (alternative schools). Child abuse is linked with school dropout (Porche et al. 2011), so these results may not be representative of all abused children.

This study also has noteworthy strengths, particularly as it relates to diversity. Age of onset of child abuse is an important, poorly understood topic across demographic groups. The study design and methods allowed our findings to be representative of the United States school enrolled population, encompassing all regions of country, urbanicity types, school sizes/types, and racial/ethnic groups.

These data show that child abuse in the United States is common and starts for many children during early childhood. Additional studies are needed to confirm our preliminary findings, and researchers should utilize various study designs to examine this topic, while remaining cognizant of the biases each method introduces. Such research is an important precursor to studying the effects of abuse on child and adult outcomes and designing effective preventive measures.

## Abbreviations

CI: Confidence interval
NVAWS: National violence against women survey
